# The global state of research in stem cells therapy for spinal cord injury (2003–2022): a visualized analysis

**DOI:** 10.3389/fnins.2024.1323383

**Published:** 2024-01-24

**Authors:** Taoyu Chen, Jiaying Zhu, Gang Wang, Jinlei Sun, Xiaofeng Ma, Lijun Tian, Meiling Zhang, Fengyan Wang, Ze Yu

**Affiliations:** ^1^Department of Orthopedics, The First Affiliated Hospital of Baotou Medical College, Inner Mongolia University of Science and Technology, Baotou, China; ^2^College of Pharmacy, China Pharmaceutical University, Nanjing, China; ^3^Department of Orthopedics, 981st Hospital of the Chinese People’s Liberation Army Joint Logistics Support Force, Chengde, China

**Keywords:** stem cell, spinal cord injury, bibliometrics, visualized study, research Frontiers

## Abstract

**Objective:**

Our study aimed to visualize the global status and frontiers in stem cell therapy for spinal cord injury by using bibliometric methodology.

**Methods:**

Publication citation information related to stem cell therapy for spinal cord injury (SCI) studies between 2003 and 2022 was retrieved from the Web of Science Core Collection database. For the visualized study, VOS viewer software and Graph Pad Prism 9.5 were used to perform bibliometric analysis of included data and publication number statistics in stem cell therapy for the SCI domain.

**Results:**

A total of 6,686 publications were retrieved. The USA and China made the highest contributions to global research with the highest number of citations and link strength. The journal Experimental Neurology ranks as the top journal, combining the publication amount and bibliometrics results. The University of Toronto, based in Canada, was the first-ranking institution. The directions of the current study could be divided into five clusters. The research of Transplantation and Regenerative Medicine and Neurosciences Mechanism Research may be the emerging frontiers in this domain.

**Conclusion:**

In summary, stem cell therapy for spinal cord injuries is poised for more valuable advances.

## Introduction

Traumatic spinal cord injury (SCI) can cause permanent sensorimotor and autonomic dysfunction, seriously affecting a patient’s autonomous activities and quality of life (World Health Organization, 2023). With a life expectancy of several decades, the frequency of SCI is between 250 and 906 cases per million (GBD 2016 Neurology Collaborators, 2019; Barbiellini Amidei et al., 2022; World Health Organization, 2023). SCI pathophysiology is one of the most complicated medical disorders, with a main and secondary phase (Ahuja et al., 2017). The current gold standard in SCI management can be summed up as timely surgery, medical care, neurorehabilitation, and lifelong care (Zipser et al., 2022). Although the death rate has decreased due to advancements in surgery and drug therapy, there are no optimal treatment strategies to repair damaged nerve cells, and long-term function rehabilitation is still subpar (Ahuja et al., 2017; Liddelow and Barres, 2017; Mohammed et al., 2019). In traumatic SCI, neuroprotective techniques such as gene therapy, cell-based treatment, and biomaterials (Ziemba and Gilbert, 2017) are used to stop secondary injury mechanisms (Ashammakhi et al., 2019; Yoon et al., 2021; Aderinto et al., 2023). Due to its ability to remyelinate denuded axons, modulate the inflammatory response, restore damaged neuronal circuits, and provide trophic support, cellular transplantation as a regenerative therapy for spinal cord injuries has attracted a lot of attention in recent decades (Liddelow and Barres, 2017; Ashammakhi et al., 2019; Srikandarajah et al., 2023). The mechanisms of stem cells in SCI can be summarized as suppressing immunity against inflammation, releasing nutritional factors to enhance neurological recovery, and promoting the regeneration of *in situ* cells (Szymoniuk et al., 2022; Xia et al., 2023). Stem cells have been shown to enhance SCI recovery in clinical trials, while clinical translation of stem cell therapy is still difficult. Sensory, motor, and neurological recovery by stem cells has been widely demonstrated (Shinozaki et al., 2021; Szymoniuk et al., 2022; Xia et al., 2023). There are various challenges that affect the progress of stem cell research, such as low patient homogeneity, small sample size, insufficient follow-up duration, insufficient understanding of SCI pathophysiology, and poor cell survival regarding cell type, dosing, and biomaterials delivery (Shang et al., 2022; Hejrati et al., 2023; Schultz et al., 2023; Wong et al., 2023). In-depth research is currently ongoing to determine the best cell type and transplantation technique for lesion bridging and remodeling, reducing immune rejection, and creating stable circuits (Zipser et al., 2022; Srikandarajah et al., 2023).

Bibliometric analysis as a method can outline data in the vast literature based on literary metrology characteristics and literature databases. This allows for the quantitative and qualitative estimation of trends in previous years’ research activity. It provides a means of identifying advancements in a specific domain and contrasting the contributions of publications, organizations, and nations (Wang et al., 2022). In recent years, bibliometric analysis has been successfully utilized in several research domains to support the creation of novel theories and has also been used in assessing research frontiers in pain management in OA (Chen et al., 2021), brain-computer interface technology (Li et al., 2023), microbiome-gut-brain axis (Zyoud et al., 2019), and COVID-19 (Goswami and Labib, 2022). A study on the same topic was published in 2019 (Guo et al., 2019), with the latest research evolving rapidly; therefore, we conducted an updated discussion of stem cell therapy for spinal cord injury, unmasking trends that may be useful for learning about several international advancements in the domain and future research frontiers.

## Methods

### Data source and search methods

Literature citation messages from the Web of Science Core Collection (WoSCC) database, deemed as an ideal and commonly used data source, were analyzed via bibliometric analysis (Leydesdorff et al., 2013). All papers were retrieved in the WoSCC from January 1, 2003 to December 31, 2022, involving the articles in the domain over the last two decades. In the present study, the search terms were as follows: (TS = (spinal cord injury) OR TS = (spinal injury) OR TS = (spinal cord trauma)) and ((TS = (stem cell)) OR TS = (stem cells)) and PY = (2003–2022) AND LA = (English). We limited the article types to original research and reviews.

### Data collection

The entire records information of all qualifying publications including title, author, year of publication, nation, affiliation, journal, keywords, and abstract were downloaded from the WOSCC. Graph Pad Prism 9.5 was used for publication number statistics.

### Bibliometric analysis

The intrinsic function of the WOS database was used to establish the basic characteristics of papers. The VOS viewer software 1.6.18 (Leiden University, Leiden, The Netherlands) was used for bibliometric visualization and analysis of the literature (van Eck and Waltman, 2010), including co-authorship, bibliographic coupling, co-citation, and co-occurrence analysis (Boyack and Klavans, 2010).

## Results

### Amount of world publication

**Figure 1:**
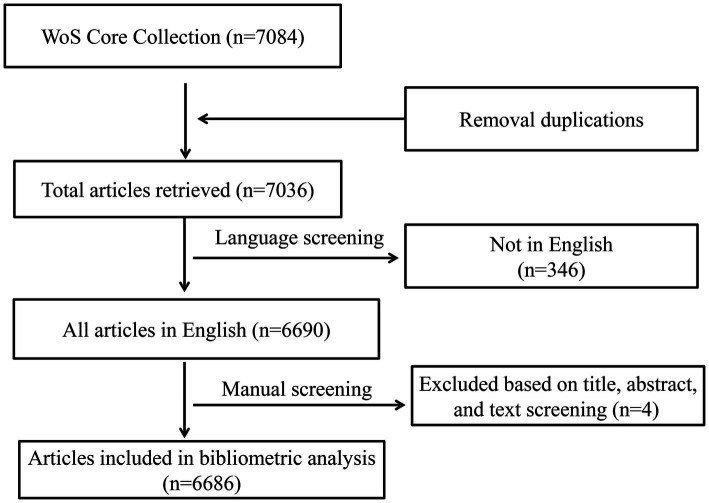
Articles’ search flow chart.

**Figure 2:**
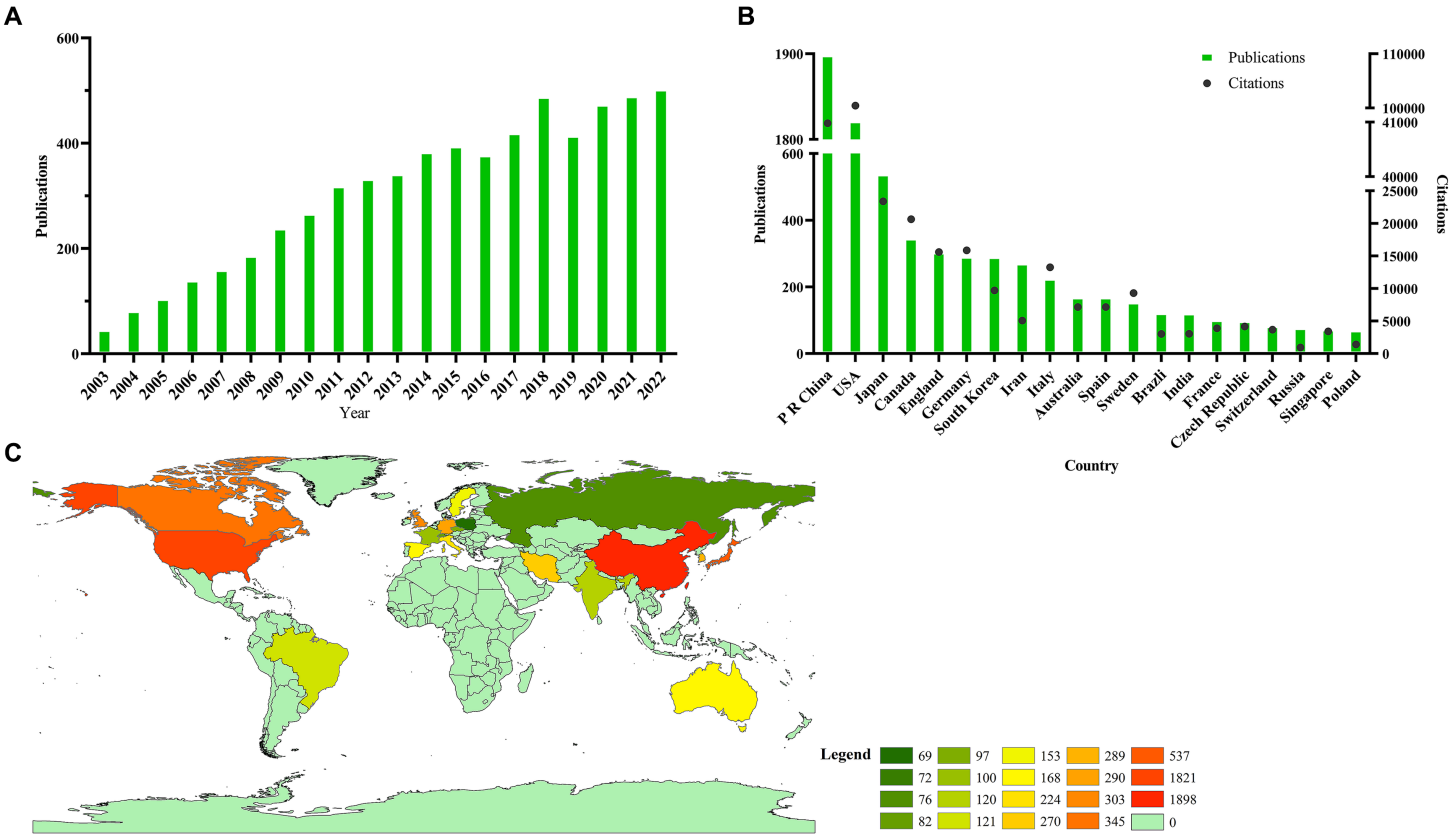
Global trends and countries contributing to stem cell therapy for spinal cord injury research. **(A)** The global number of publications related to stem cell therapy for spinal cord injury research. The green bars indicate the single-year publication numbers. **(B)** The sum of stem cell therapy for spinal cord injury research-related articles from the top 20 countries. The green bars indicate the single-country publication number, and the black spot indicates the citation number of every country. **(C)** World map showing the distribution of stem cell therapy for spinal cord injury research.

From 2003 to 2022, a total of 6,686 articles met the search criteria. The process for the selection and inclusion of the title catalog is illustrated in Figure 1. By measuring the publication time and trend distribution, the number of publications peaked in 2018 with 488 literature and fell to 414 in 2019. From 2019 to 2022, a sluggish rise in worldwide publications was seen (Figure 2A).

### Publication distribution across nations

A total of 81 nations and regions contributed to this domain. China published the most related articles out of all of these nations (1,898, 30.83%), followed by the USA (1,821, 29.62%), Japan (537, 8.74%), Canada (345, 5.61%), and England (303, 4.93%). The top 20 countries are shown in a bar chart and color-coded on the world map (Figures 2B,C).

### Total citation frequency

The number of citations for publications from the USA was the greatest (100,441), while China ranked second (40,982), followed by Japan (23,388), Canada (20,637), and Germany (15,873) (Figure 2B).

### Analysis of world publication

#### Publication distribution across journal

**Figure 3:**
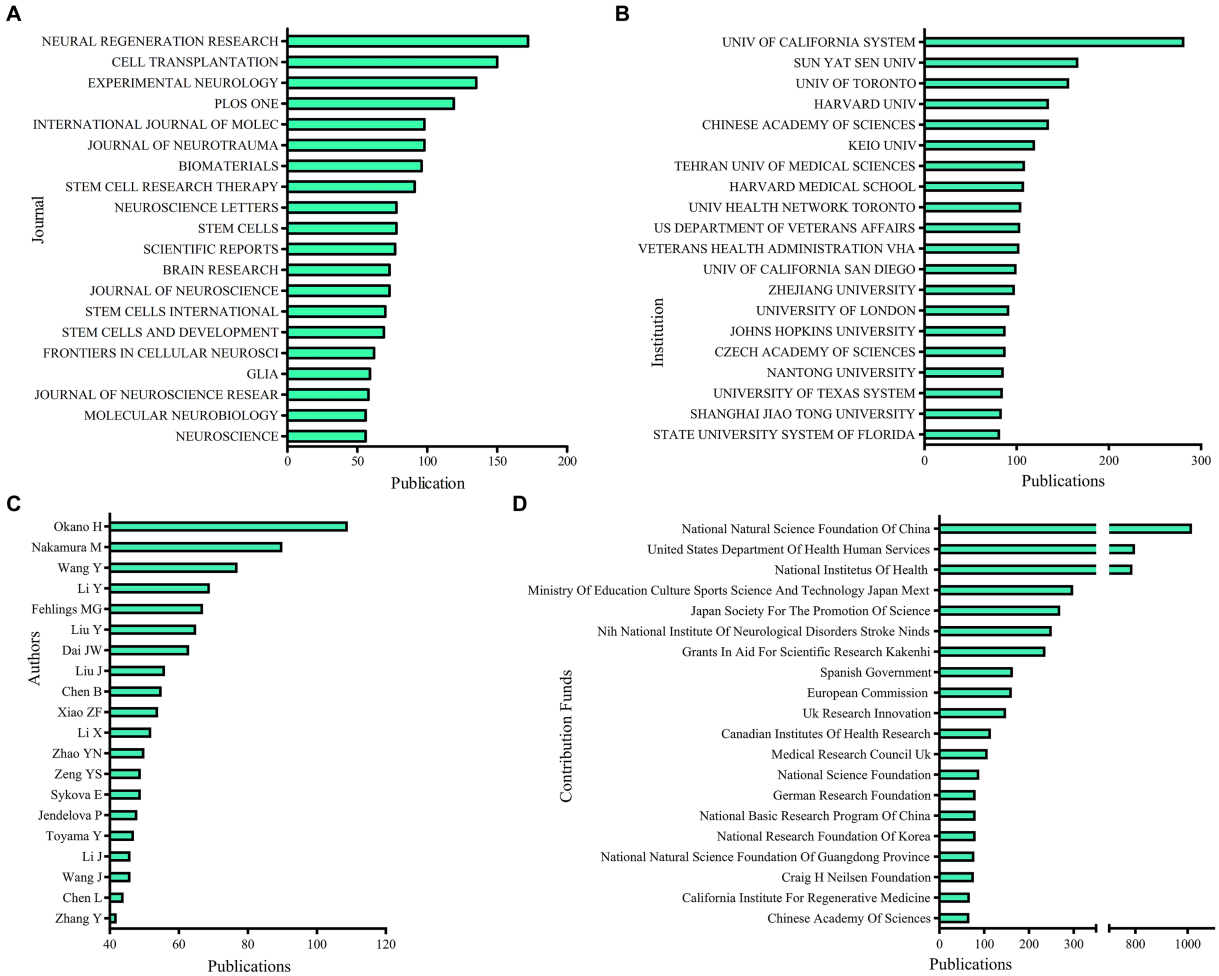
Publication amounts of different journals, institutions, authors, and contribution funds. **(A)** The sum of stem cell therapy for spinal cord injury research from the top 20 journals. **(B)** The sum of stem cell therapy for spinal cord injury research from the top 20 institutions. **(C)** The sum of stem cell therapy for spinal cord injury research from the top 20 authors. **(D)** The sum of stem cell therapy for spinal cord injury research from the top 20 contribution funds.

The journal Neural Regeneration Research published the most studies with 172 publications. There were 150 articles in Cell Transplantation, 135 articles in Experimental Neurology, 119 articles in PLoS One, and 98 articles in the International Journal of Molecular Sciences on stem cell therapy for spinal cord injury. Table 1 lists the top 10 journals by the number of studies with their Quartile in Category (2022), and the top 20 journals are shown in a bar chart (Figure 3A).

**Table 1:** The top 10 journals with most published literature from 2003 to 2022.

Ranking	Journal	Publications	Times cited	Times cited (per article)	Periodical Division of the Documentation and Information Center of the Chinese Academy of Sciences (2022)
1	Neural Regeneration Research	172	2,534	14.7	Q2
2	Cell Transplantation	150	5,318	35.5	Q4
3	Experimental Neurology	135	8,571	63.5	Q2
4	PLoS One	119	5,028	42.3	Q3
5	International Journal of Molecular Sciences	98	2,331	23.8	Q2
6	Journal of Neurotrauma	98	4,405	44.9	Q2
7	Biomaterials	96	7,101	74.0	Q1
8	Stem Cell Research and Therapy	91	2,740	30.1	Q2
9	Neuroscience Letters	78	2,370	30.4	Q4
10	Stem Cells	78	6,718	86.1	Q2

#### Publication distribution across institutions

Figure 3B lists the top 20 producing institutions. The University of California System published the largest number of articles (282), Sun Yat-sen University came in second (167), the University of Toronto came in third (157), followed by the Chinese Academy of Sciences (135), and Harvard University (135).

#### Publication distribution across authors

The top 10 authors contributed to 570 papers in total or 8.53% of all publications in this subject (Table 2). Okano Hideyuki and Nakamura Masaya ranking first and second, respectively, are both from Keio University in Japan. Dai Jianwu, Xiao Zhifeng, Zhao Yannan, and Chen Bing had a close cooperation in China. Figure 3C displays the top 20 authors as a bar chart.

**Table 2:** The top 10 active authors with most publications from 2003 to 2022.

Ranking	Author	Publications	Times cited	Times cited (per article)	Institution
1	Okano Hideyuki	97	5,897	60.8	Keio University
2	Nakamura Masaya	77	4,270	55.5	Keio University
3	Fehlings Michael G.	64	5,138	80.3	University of Toronto
4	Dai Jianwu	62	2,299	37.1	Chinese Academy of Sciences
5	Xiao Zhifeng	53	1,971	37.2	Army Medical University
6	Zhao Yannan	49	1,729	35.3	Chinese Academy of Sciences
7	Chen Bing	44	1,588	36.1	Chinese Academy of Sciences
8	Sykova Eva	43	2,619	60.9	Czech Academy of Sciences
9	Zeng Yuanshan	41	1,431	34.9	Sun Yat-sen University
10	Jendelova Pavla	40	1,942	48.6	Charles University

### Contribution funds across WoS categories

In total, the top 20 major funds across WoS categories have supported 5,311 research as shown in Figure 3D. National Natural Science Foundation of China (NSFC), the United States Department of Health and Human Services, and the National Institutes of Health were the top three fund sources, supporting 1,017, 799, and 789, respectively. Two Japanese funds ranked fourth and fifth, with the Ministry of Education Culture Sports Science and Technology Japan Mext (299) and Japan Society for The Promotion of Science (270), respectively.

### Co-authorship analysis

Co-authorship analysis measures researchers’ publication links, which can be used to examine the link strength of individual authors or scaled up to reflect the co-authorship link strength of nations and institutions (Chen et al., 2021).

**Figure 4:**
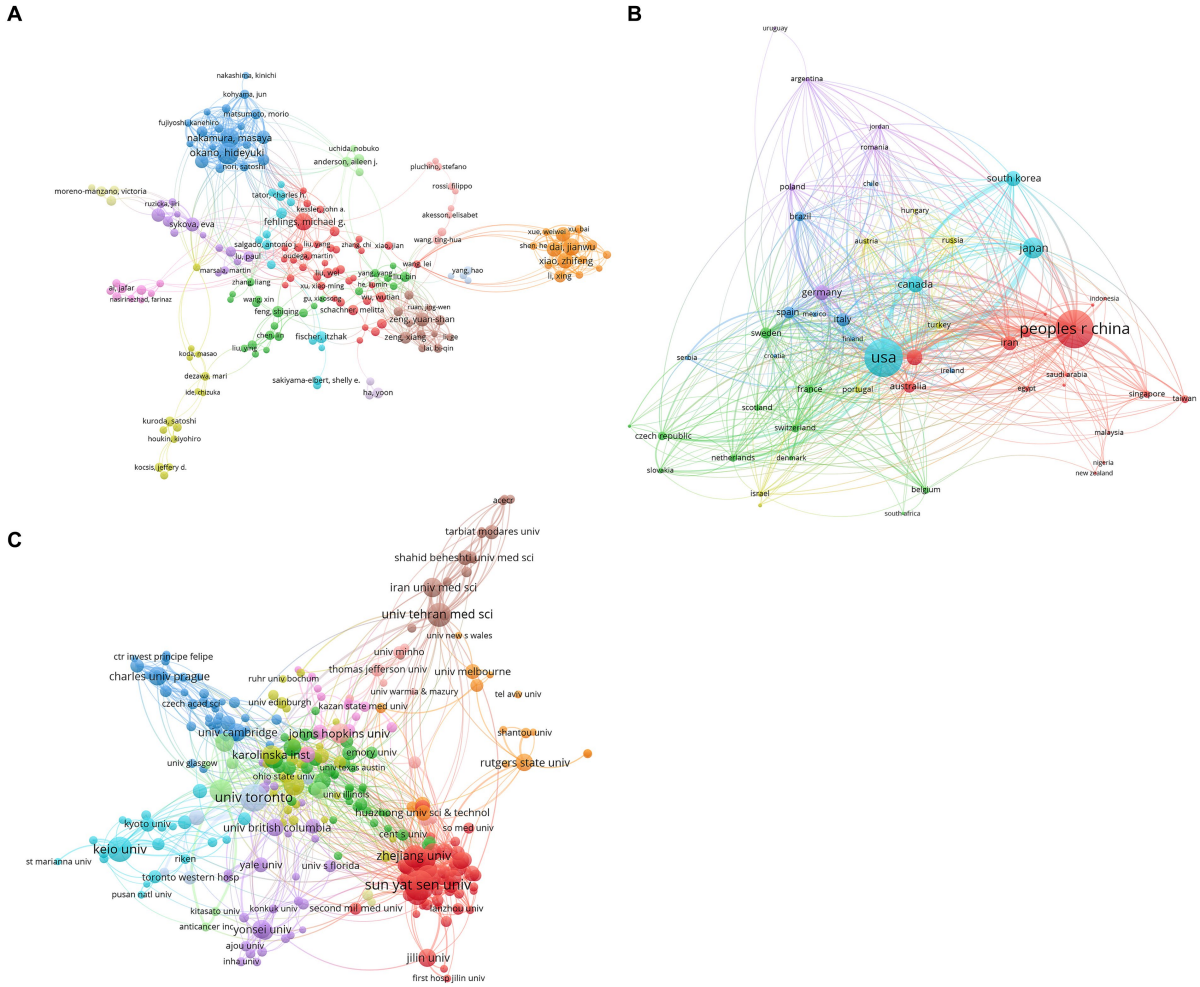
Co-authorship analysis of stem cell therapy for spinal cord injury research. **(A)** Mapping of the 209 authors’ co-authorship analysis on stem cell therapy for spinal cord injury research. **(B)** Mapping of the 53 countries’ co-authorship analysis on stem cell therapy for spinal cord injury research. **(C)** Mapping of the 294 institutions’ co-authorship analysis on stem cell therapy for spinal cord injury research. The size of the points represents that two authors/countries/institutions had established collaboration. The thicker the line, the closer the link between the two authors/countries/institutions.

According to co-authorship analysis, the relatedness of items is based on the number of papers co-authored, and 209 authors that published 10 articles or more were examined (Figure 4A). The following were the top five authors with strong link strength: Okano Hideyuki (579); Nakamura Masaya (553); Dai Jianwu (446); Xiao Zhifeng (419); Zhao Yannan (396).

VOS viewer was used to evaluate 53 countries whose publications were five or more (Figure 4B). The USA had a total link strength of 1,102, followed by China with 533, England with 340, Germany with 328, and Japan with 263..

Publications found in the 294 academic affiliations whose publications were five or more were examined (Figure 4C). University of Toronto (197), the University of California, San Diego (184), Sun Yat-sen University (179), Tehran University of Medical Sciences (167), and Chinese Academy of Sciences (149) were the top five universities with high total link strength.

### Bibliographic coupling analysis

**Figure 5:**
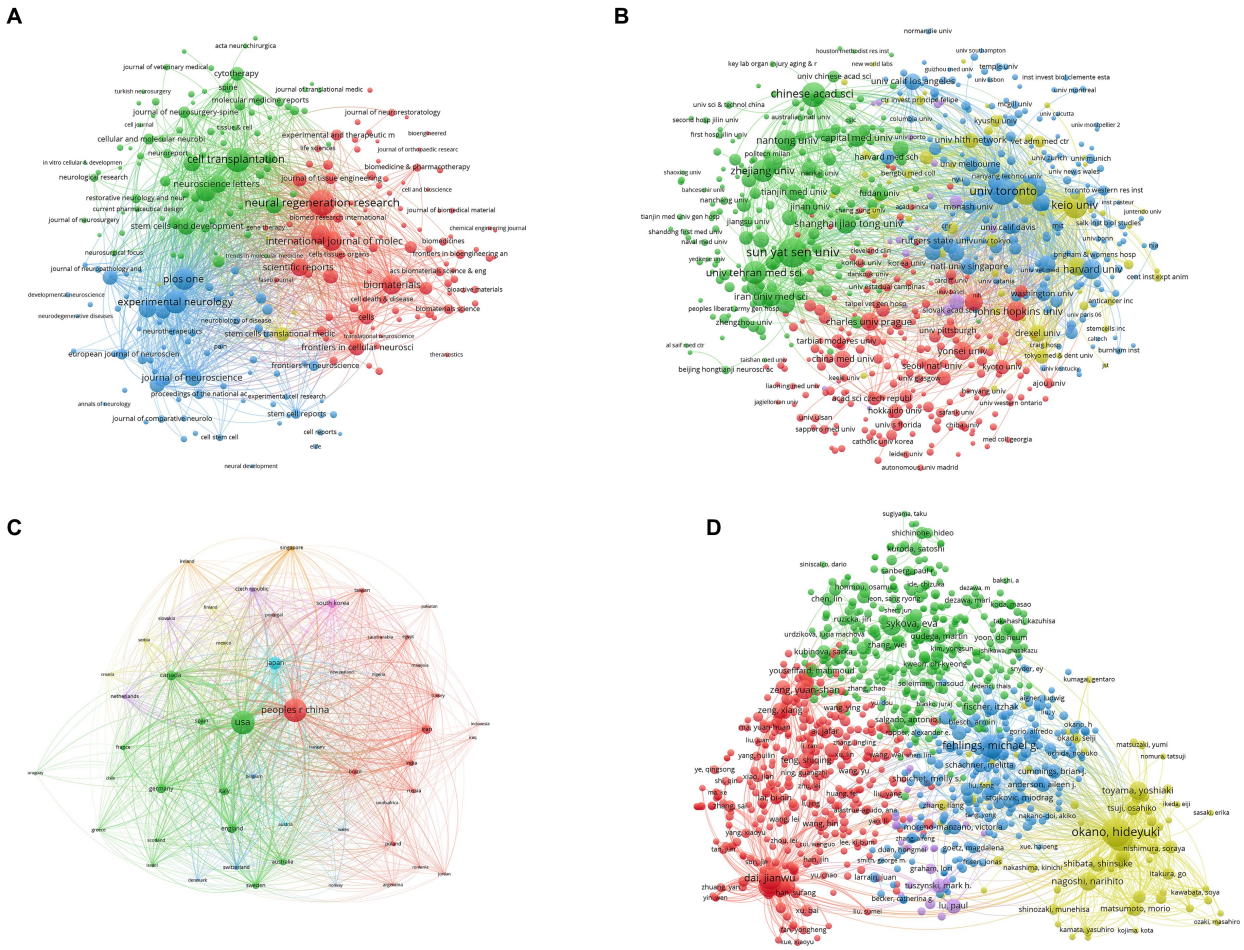
Bibliographic coupling analysis of stem cell therapy for spinal cord injury research. **(A)** Mapping of the 250 identified journals on stem cell therapy for spinal cord injury research. **(B)** Mapping of the 639 identified institutions on stem cell therapy for spinal cord injury research. **(C)** Mapping of the 53 identified countries on stem cell therapy for spinal cord injury research. **(D)** Mapping of the 984 identified authors on stem cell therapy for spinal cord injury research. The line between the two journals/institutions/countries shows that they had established a similarity relationship. The thicker the line, the closer the link between the two journals/institutions/countries/authors.

Using VOS viewer, the names of the journals in all articles were examined. A total of 250 recognized journals were visible in the link strength, as seen in Figure 5A. Cell Transplantation (256,384), Experimental Neurology (230,171), Journal of Neurotrauma (182,712), Neural Regeneration Research (170,468), and PLoS One (151,956) were the top five journals with the highest total link strength.

Papers found in the 639 institutions whose publications were five or more were examined. University of Toronto (790,059), Keio University (472,486), Sun Yat-sen University (415,185), Chinese Academy of Sciences (335,691), and the University of California, San Diego (307,960), were the top five universities with the highest total link strength (Figure 5B).

Papers found in the 53 countries whose publications were five or more were examined. USA (3,048,437), China (2,393,090), Japan (1,104,477), Canada (1,067,438), and England (712,587) were the top five countries with the highest total link strength (Figure 5C).

The authors among the 984 that published 10 articles or more were examined. The top five authors with the strongest total link strength were Okano Hideyuki (2,462,776); Nakamura Masaya (1,033,960); Fehlings Michael G. (928,593); Dai Jianwu (716,183); Xiao Zhifeng (561,444) (Figure 5D).

### Co-citation analysis

The relationship between things based on how frequently they were quoted in a single document is displayed through co-citation analysis. The overall co-citation connection strength of authors or journals was examined using the VOS viewer (van Eck and Waltman, 2017).

**Figure 6:**
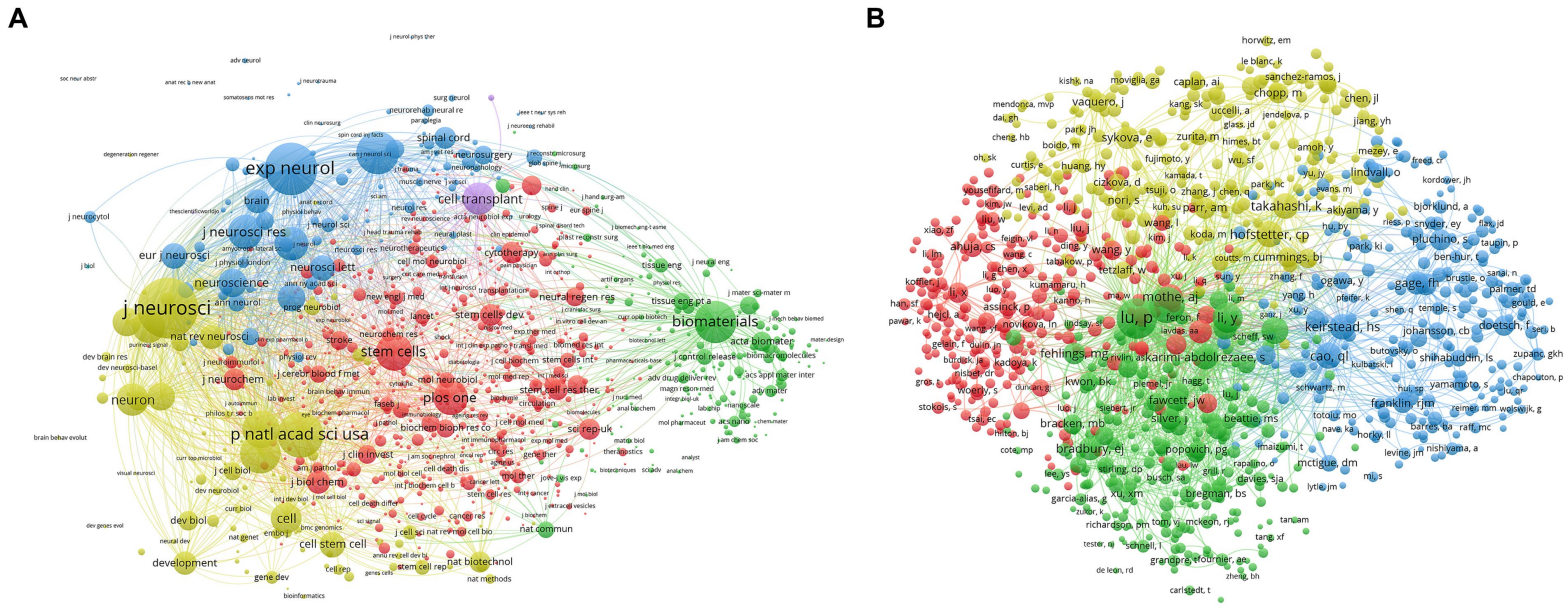
Co-citation analysis of stem cell therapy for spinal cord injury research. **(A)** Mapping of the 1,000 journals’ co-citation analysis on stem cell therapy for spinal cord injury research. **(B)** Mapping of the 1,000 authors’ co-citation analysis on stem cell therapy for spinal cord injury research. The size of the points represents that two journals/authors had established collaboration. The thicker the line, the closer the link between the two journals/authors.

A total of 1,000 journals’ link strength was displayed, and every journal that was chosen had at least 38 co-citations in this domain. The following were the top five journals with a high total link strength: 2,032,080 times in the Journal of Neuroscience; 1,397,751 times in Experimental Neurology; 1,143,804 times in the Proceeding of the National Academy of Sciences USA; 964,548 times in Nature; and 964,462 times in Biomaterials (Figure 6A).

A total of 1,000 authors’ link strengths were displayed, and every chosen author had at least 55 co-citations in this area. The following authors ranked in the top five with strong overall links: Lu P. (61,747), Li Y. (32,781), Cao Q. L. (29,387), Basso D. M. (27,985), and Mcdonal J. W. (27,438) are the other five individuals with total link strengths are shown in Figure 6B.

### Co-occurrence analysis

**Figure 7:**
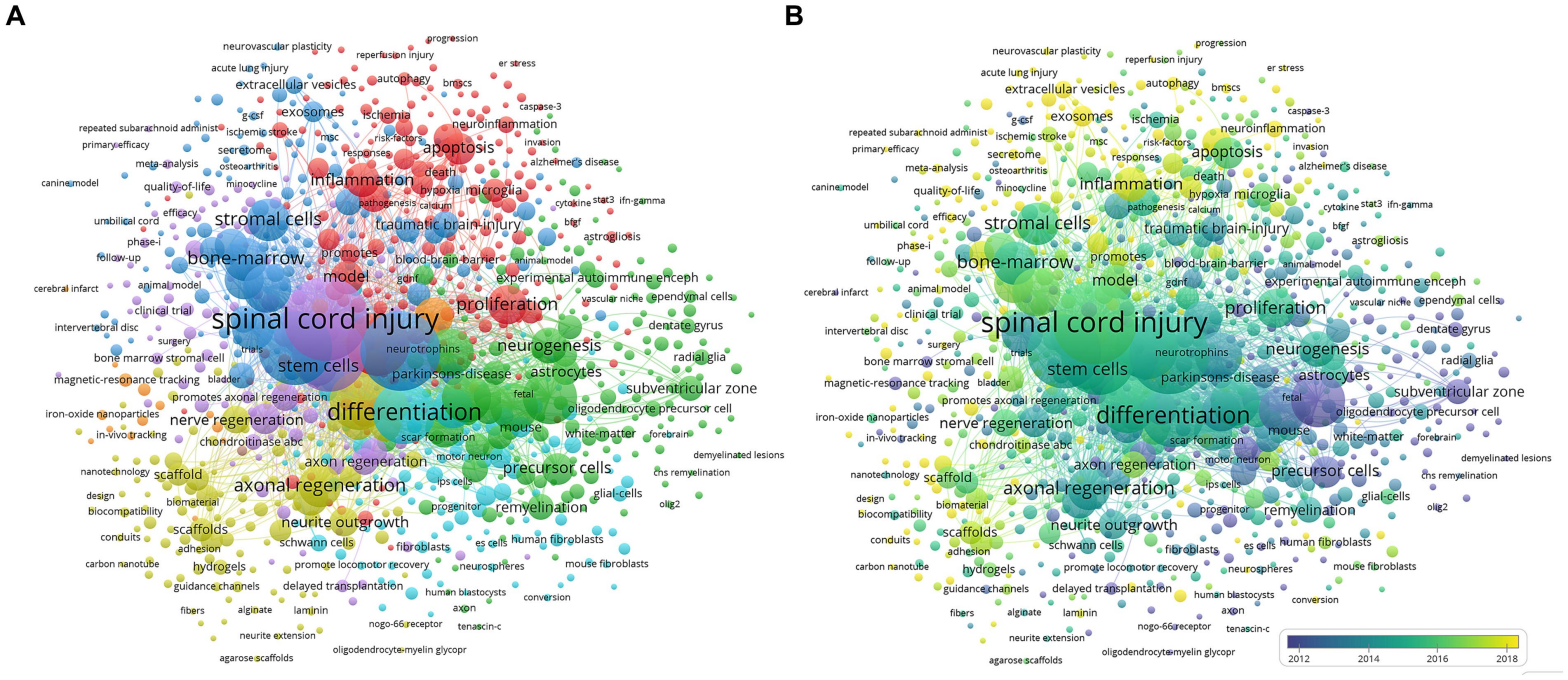
Co-occurrence analysis of research of stem cell therapy for spinal cord injury. **(A)** Mapping of keywords in the research about stem cell therapy for spinal cord injury; the size of the points represents the frequency, and the keywords are divided into five clusters: Neurosciences Mechanism Research (upper in red), Clinical Therapy (left in purple), Combinatorial Therapy (left in blue), Types of Stem Cell (right in green), and Transplantation and Regenerative Medicine (lower in yellow). **(B)** Distribution of keywords according to the timeline of appearance; keywords in blue appeared earlier than those in yellow.

The purpose of co-occurrence analysis is to discover research interests and emerging topics in literature, and it has proven to be important for monitoring the development of science and programs (van Eck and Waltman, 2009; Wang et al., 2019). Keywords that appeared five times or more were analyzed using VOS viewer. Node size in the figure indicates the frequency of occurrence, and lines represent connections between nodes. As shown in Figure 7A, the 1,000 identified keywords were classified into approximately five clusters: Combinatorial Therapy; Types of Stem Cells; Clinical Therapy, Transplantation, and Regenerative Medicine; and Neurosciences Mechanism Research.

On the other hand, the timeline graph in Figure 7B shows the chronological distribution of the keywords. The blue color indicates that the keyword appeared earlier and the yellow color keywords appeared later. Before 2012, namely, in the early stage of research, most studies focused on Types of Stem Cells. The latest trends showed that the Transplantation and Regenerative Medicine and Neurosciences Mechanism Research clusters would be concerned widely in the future.

## Discussion

In this study, we used a combination of bibliometric and visualized analyses to generate a representation of the current state of stem cell therapy for spinal cord injury. We have analyzed the amount of global publication volume and the relative contributions of journals, authors, institutions, and contribution funds to this field.

### Research trends analysis

Since 2003, the publication number has seen continued growth until it peaked in 2018, and it recovered to the 2018 levels by 2022, indicating increasing attention from scholars. The advent of new technology, such as spatiotemporal epidural electrical stimulation (Kathe et al., 2022) and brain-spine interface (Lorach et al., 2023), diverting research interests may be the reason for the decline in articles. In addition, stem cell therapies for SCI not having yet provided reproducible evidence may be another reason, challenged by small effect sizes, low immune suppression, and low sensitivity study design (Zipser et al., 2022; Hejrati et al., 2023; Wong et al., 2023).

### Quality of global publications by country, author, institution, and journal

China has the highest number of publications and the second total citation frequency, while the USA has a smaller amount of literature, and the total citation frequency is almost twice that of China. The top two countries have the largest number of fund supports, as well as the top rank for bibliographic coupling and co-authorship analyses conducted by country. These trends suggest that the USA and China have the largest quantity, highest academic impact, and extensive cooperation in this field. With increases in Chinese research funding, the quality of publications and academic impact from Chinese academia should be further improved. On the other hand, Japan and South Korea in Asia and England, Germany, and Italy in Europe have had a large amount of publication, quality, and impact over the past two decades.

The relative contributions of specific institutions to the field of stem cell therapy for SCI were reflected in publication amount and link strengths of bibliographic coupling and co-authorship analyses. Not unexpectedly, the highest contributing institutions are from top contributing countries, particularly the USA and China. University of Toronto, based in Canada, is the first-ranking institution. The University of California San Diego, Sun Yat-sen University, and the Chinese Academy of Sciences are the top-class institutions. It is worth mentioning that the Tehran University of Medical Sciences, based in Iran, is the only institution that does not belong in the Middle East. The color cluster results show that the top class institutions in the same country are highly collaborative and interconnected.

Okano Hideyuki and Nakamura Masaya, committed to research in induced pluripotent stem (iPS) cells to repair a spinal cord injury, are both from Keio University, another top-class institution in this field in Japan. Fehlings Michael G. is another author with a high publication volume and citation from the University of Toronto. Dai Jianwu, Xiao Zhifeng, Zhao Yannan, and Chen Bing had a close cooperation from China. Authors and their institutions can contribute significantly to this field and win great influence. According to clusters, within same color group, the cooperation of the author and institution are tight. On the other hand, the different color groups cooperated more loosely. Therefore, closer academic cooperation between different groups of countries and institutions may yield more achievements.

The relative contributions of journals to the field of stem cell therapy for SCI were reflected in publication amount and link strengths of bibliographic coupling and co-citation analyses. Combining the publication amount and bibliometrics results, Experimental Neurology ranks as the top journal, with 135 articles published, cited 8,571 times, according to periodical division area 2 of the Documentation and Information Center of the Chinese Academy of Sciences. Cell Transplantation, Neural Regeneration Research, Journal of Neuroscience, and Proceeding of the National Academy of Sciences USA are called top co-cited journals. These journals may become a platform mainly publishing research in stem cell therapy for SCI and win more attention.

## Future outlook

Future directions in stem cell therapy for spinal cord injury are indicated by co-occurrence network maps clustered by keywords area or timeline. Research directions in this field were divided into Combinatorial Therapy; Types of Stem Cell; Clinical Therapy, Transplantation, and Regenerative Medicine; and Neurosciences Mechanism Research. Several terms colored toward the yellow end of the spectrum, indicating more recent publication dates, belong under the Transplantation and Regenerative Medicine and Neurosciences Mechanism Research clusters, suggesting these research topics will continue to be hot.

SCI is still a tough challenge mainly due to various pathological mechanisms including hemorrhage, ischemia, oxidative stress, inflammatory reaction, scar formation, and demyelination, which are difficult to clearly describe and elaborate (Kim et al., 2011; Ashammakhi et al., 2019; Aderinto et al., 2023; Hu et al., 2023). The cell response is the basic unit in the pathophysiology of SCI. The elaboration of stem cell response mechanisms is of great importance for finding effective intervention targets for SCI. Stem cells derive from a wide range of sources and have self-proliferation and multidirectional differentiation capabilities (Liu et al., 2022). The immunomodulatory mechanism is the most attractive aspect, mediated by contact between stem cells and immune cells depending on the realization of exosomes produced by the paracrine effect (Ankrum et al., 2014). Another mechanism is the promotion of axon regeneration to repair the damaged cells. In addition, stem cells can promote vascular repair, which is a new target for SCI treatment (Ni et al., 2018). When the understanding of the molecular mechanism is sufficient, we can find reliable strategies to boost stem cells’ functional multipotency (Feng et al., 2022).

To achieve better treatment of SCI with stem cells, transplantation and regenerative medicine are needed, which is a combination of stem cells and biomaterials via tissue engineering (Aderinto et al., 2023). Stem cell transplantation has been deemed to be a promising way to replenish the lost spinal nerve cells (Xu et al., 2023). As mentioned above, the effectiveness of stem cell injection is hampered by challenges in cell delivery and low cell survival rates, while co-transplantation of stem cells and biological scaffold may have the potential to improve treatment performance but can lead to adverse reactions, including local inflammation and immune rejection (Chen et al., 2021). Regenerative medicine currently focuses on the aspects of 3-dimensional network to preserve the stem cell at the site of injury, extracellular matrix better maintaining cell viability, and biological strength. In the future, neurosciences mechanism, transplantation, and regenerative medicine still need more in-depth research (Yousefifard et al., 2016; Wallace et al., 2019; Zhang et al., 2022).

### Strengths and limitations

Although the present study evaluated the overall situation and trend of stem cell therapy for spinal cord injury via bibliometric and visualized analyses, the following items about limitations have to be mentioned. English language articles and reviews were included based on the SCIE database of WOS. Non-English language literature could have been omitted, leading to language bias. Additionally, differences may exist between the real world and the present results. Therefore, we still need to focus on the latest primary studies and other non-English studies in our daily research work.

## Conclusion

The present study showed the global trend in stem cell therapy for spinal cord injury. The USA and China are the top two contributors to studies and have the leading position in global research in this field. The journal Experimental Neurology had the most publications related to this issue. We believe that more studies about stem cell therapy for spinal cord injury will be published in the coming years. Particularly, the Transplantation and Regenerative Medicine and Neurosciences Mechanism Research studies, involving stem cell therapy for spinal cord injury, are the next popular hot spots.

## Author contributions

TC: Visualization, Writing – original draft, Writing – review & editing. JZ: Writing – original draft. GW: Writing – original draft. JS: Writing – original draft. XM: Writing – original draft. ZY: Funding acquisition, Writing – original draft. LT: Reporting and editing. FW: Final approval of the version to be submitted. MZ: Final approval of the version to be submitted.

## References

[ref1] Aderinto N. Abdulbasit M. O. Olatunji D. (2023). Stem cell-based combinatorial therapies for spinal cord injury: a narrative review of current research and future directions. Ann. Med. Surg. 85, 3943–3954. doi: 10.1097/MS9.0000000000001034 PMC10406006 37554849

[ref2] Ahuja C. S. Wilson J. R. Nori S. Kotter M. R. N. Druschel C. Curt A.. (2017). Traumatic spinal cord injury. Nat. Rev. Dis. Primers 3:17018. doi: 10.1038/nrdp.2017.18 28447605

[ref3] Ankrum J. A. Ong J. F. Karp J. M. (2014). Mesenchymal stem cells: immune evasive, not immune privileged. Nat. Biotechnol. 32, 252–260. doi: 10.1038/nbt.2816, PMID: 24561556 PMC4320647

[ref4] Ashammakhi N. Kim H. J. Ehsanipour A. Bierman R. D. Kaarela O. Xue C.. (2019). Regenerative therapies for spinal cord injury. Tissue Eng. B 25, 471–491. doi: 10.1089/ten.teb.2019.0182, PMID: 31452463 PMC6919264

[ref5] Barbiellini Amidei C. Salmaso L. Bellio S. Saia M. (2022). Epidemiology of traumatic spinal cord injury: a large population-based study. Spinal Cord 60, 812–819. doi: 10.1038/s41393-022-00795-w, PMID: 35396455 PMC8990493

[ref6] Boyack K. W. Klavans R. (2010). Co-citation analysis, bibliographic coupling, and direct citation: which citation approach represents the research front most accurately? J. Am. Soc. Inf. Sci. Technol. 61, 2389–2404. doi: 10.1002/asi.21419

[ref7] Chen X. Wang Y. Zhou G. Hu X. Han S. Gao J. (2021). The combination of nanoscaffolds and stem cell transplantation: paving a promising road for spinal cord injury regeneration. Biomed. Pharmacother. 143:112233. doi: 10.1016/j.biopha.2021.112233, PMID: 34649357

[ref8] Chen T. Zhu J. Zhao Y. Li H. Li P. Fan J.. (2021). The global state of research in pain management of osteoarthritis (2000–2019): a 20-year visualized analysis. Medicine 100:e23944. doi: 10.1097/MD.0000000000023944, PMID: 33466135 PMC7808549

[ref9] Feng Y. Li Y. Shen P.-P. Wang B. (2022). Gene-modified stem cells for spinal cord injury: a promising better alternative therapy. Stem Cell Rev. Rep. 18, 2662–2682. doi: 10.1007/s12015-022-10387-z, PMID: 35587330

[ref10] GBD 2016 Neurology Collaborators (2019). Global, regional, and national burden of neurological disorders, 1990–2016: a systematic analysis for the Global Burden of Disease Study 2016. Lancet Neurol. 18, 459–480. doi: 10.1016/S1474-4422(18)30499-X 30879893 PMC6459001

[ref11] Goswami G. Labib T. (2022). Modeling COVID-19 transmission dynamics: a bibliometric review. Int. J. Environ. Res. Public Health 19:14143. doi: 10.3390/ijerph192114143, PMID: 36361019 PMC9655715

[ref12] Guo S. Wang L. Xie Y. Luo X. Zhang S. Xiong L.. (2019). Bibliometric and visualized analysis of stem cells therapy for spinal cord injury based on web of science and cite space in the last 20 years. World Neurosurg. 132, e246–e258. doi: 10.1016/j.wneu.2019.08.191, PMID: 31493613

[ref13] Hejrati N. Wong R. Khazaei M. Fehlings M. G. (2023). How can clinical safety and efficacy concerns in stem cell therapy for spinal cord injury be overcome? Expert. Opin. Biol. Ther. 23, 883–899. doi: 10.1080/14712598.2023.2245321, PMID: 37545020

[ref14] Hu X. Xu W. Ren Y. Wang Z. He X. Huang R.. (2023). Spinal cord injury: molecular mechanisms and therapeutic interventions. Signal Transduct. Target. Ther. 8:245. doi: 10.1038/s41392-023-01477-6, PMID: 37357239 PMC10291001

[ref15] Kathe C. Skinnider M. A. Hutson T. H. Regazzi N. Gautier M. Demesmaeker R.. (2022). The neurons that restore walking after paralysis. Nature 611, 540–547. doi: 10.1038/s41586-022-05385-7, PMID: 36352232 PMC9668750

[ref16] Kim S. H. Turnbull J. Guimond S. (2011). Extracellular matrix and cell signalling: the dynamic cooperation of integrin, proteoglycan and growth factor receptor. J. Endocrinol. 209, 139–151. doi: 10.1530/JOE-10-0377, PMID: 21307119

[ref17] Leydesdorff L. Carley S. Rafols I. (2013). Global maps of science based on the new web-of-science categories. Scientometrics 94, 589–593. doi: 10.1007/s11192-012-0784-8, PMID: 23335826 PMC3547244

[ref18] Li F. Zhang D. Chen J. Tang K. Li X. Hou Z. (2023). Research hotspots and trends of brain-computer interface technology in stroke: a bibliometric study and visualization analysis. Front. Neurosci. 17:1243151. doi: 10.3389/fnins.2023.1243151 37732305 PMC10507647

[ref19] Liddelow S. A. Barres B. A. (2017). Reactive astrocytes: production, function, and therapeutic potential. Immunity 46, 957–967. doi: 10.1016/j.immuni.2017.06.006 28636962

[ref20] Liu J. Gao J. Liang Z. Gao C. Niu Q. Wu F.. (2022). Mesenchymal stem cells and their microenvironment. Stem Cell Res Ther 13:429. doi: 10.1186/s13287-022-02985-y, PMID: 35987711 PMC9391632

[ref21] Lorach H. Galvez A. Spagnolo V. Martel F. Karakas S. Intering N.. (2023). Walking naturally after spinal cord injury using a brain-spine interface. Nature 618, 126–133. doi: 10.1038/s41586-023-06094-5, PMID: 37225984 PMC10232367

[ref22] Mohammed J. Aljurf M. Althumayri A. Almansour M. Alghamdi A. Hamidieh A. A.. (2019). Physical therapy pathway and protocol for patients undergoing hematopoietic stem cell transplantation: recommendations from The Eastern Mediterranean Blood and Marrow Transplantation (EMBMT) Group. Hematol. Oncol. Stem Cell Ther. 12, 127–132. doi: 10.1016/j.hemonc.2018.12.003, PMID: 30653940

[ref23] Ni S. Cao Y. Jiang L. Luo Z. Lu H. Hu J.. (2018). Synchrotron radiation imaging reveals the role of estrogen in promoting angiogenesis after acute spinal cord injury in rats. Spine 43, 1241–1249. doi: 10.1097/BRS.0000000000002629, PMID: 29529001

[ref24] Schultz J. L. Neema M. Nopoulos P. C. (2023). Unravelling the role of huntingtin: from neurodevelopment to neurodegeneration. Brain J. Neurol. 146, 4408–4410. doi: 10.1093/brain/awad353, PMID: 37816304 PMC10629758

[ref25] Shang Z. Wang R. Li D. Chen J. Zhang B. Wang M.. (2022). Spinal cord injury: a systematic review and network meta-analysis of therapeutic strategies based on 15 types of stem cells in animal models. Front. Pharmacol. 13:819861. doi: 10.3389/fphar.2022.819861, PMID: 35359872 PMC8964098

[ref26] Shinozaki M. Nagoshi N. Nakamura M. Okano H. (2021). Mechanisms of stem cell therapy in spinal cord injuries. Cells 10:2676. doi: 10.3390/cells10102676, PMID: 34685655 PMC8534136

[ref27] Srikandarajah N. Alvi M. A. Fehlings M. G. (2023). Current insights into the management of spinal cord injury. J. Orthop. 41, 8–13. doi: 10.1016/j.jor.2023.05.007, PMID: 37251726 PMC10220467

[ref28] Szymoniuk M. Litak J. Sakwa L. Dryla A. Zezuliński W. Czyżewski W.. (2022). Molecular mechanisms and clinical application of multipotent stem cells for spinal cord injury. Cells 12:120. doi: 10.3390/cells12010120, PMID: 36611914 PMC9818156

[ref29] van Eck N. J. Waltman L. (2009). How to normalize cooccurrence data? An analysis of some well-known similarity measures. J. Am. Soc. Inf. Sci. Technol. 60, 1635–1651. doi: 10.1002/asi.21075

[ref30] van Eck N. J. Waltman L. (2010). Software survey: VOS viewer, a computer program for bibliometric mapping. Scientometrics 84, 523–538. doi: 10.1007/s11192-009-0146-3, PMID: 20585380 PMC2883932

[ref31] van Eck N. J. Waltman L. (2017). Citation-based clustering of publications using CitNetExplorer and VOSviewer. Scientometrics 111, 1053–1070. doi: 10.1007/s11192-017-2300-7, PMID: 28490825 PMC5400793

[ref32] Wallace D. J. Sayre N. L. Patterson T. T. Nicholson S. E. Hilton D. Grandhi R. (2019). Spinal cord injury and the human microbiome: beyond the brain-gut axis. Neurosurg. Focus 46:E11. doi: 10.3171/2018.12.FOCUS18206, PMID: 30835680

[ref33] Wang N. Chen S. Zhang X. Xi Z. Fang X. Xue C.. (2022). Global research status and hot trends in stem cells therapy for intervertebral disc degeneration: a bibliometric and clinical study analysis. Front. Pharmacol. 13:873177. doi: 10.3389/fphar.2022.873177, PMID: 36003512 PMC9393636

[ref34] Wang K. Xing D. Dong S. Lin J. (2019). The global state of research in nonsurgical treatment of knee osteoarthritis: a bibliometric and visualized study. BMC Musculoskelet. Disord. 20:407. doi: 10.1186/s12891-019-2804-9, PMID: 31484517 PMC6727547

[ref35] Wong R. Hejrati N. Fehlings M. G. (2023). Clinical trials for neuroregenerative therapies for spinal cord injury: what have we learnt so far? Expert. Rev. Neurother. 23, 487–499. doi: 10.1080/14737175.2023.2215429, PMID: 37231735

[ref36] World Health Organization. Fact sheet: spinal cord injury. Available at: https://www.who.int/news-room/fact-sheets/detail/spinalcord-injury. (2023)

[ref37] Xia Y. Zhu J. Yang R. Wang H. Li Y. Fu C. (2023). Mesenchymal stem cells in the treatment of spinal cord injury: mechanisms, current advances and future challenges. Front. Immunol. 14:1141601. doi: 10.3389/fimmu.2023.1141601, PMID: 36911700 PMC9999104

[ref38] Xu L. Zhao H. Yang Y. Xiong Y. Zhong W. Jiang G.. (2023). The application of stem cell sheets for neuronal regeneration after spinal cord injury: a systematic review of pre-clinical studies. Syst. Rev. 12:225. doi: 10.1186/s13643-023-02390-3, PMID: 38037129 PMC10688065

[ref39] Yoon S. H. Bae M. R. La H. Song H. Hong K. Do J. T. (2021). Efficient generation of neural stem cells from embryonic stem cells using a three-dimensional differentiation system. Int. J. Mol. Sci. 22:8322. doi: 10.3390/ijms22158322, PMID: 34361088 PMC8348082

[ref40] Yousefifard M. Rahimi-Movaghar V. Nasirinezhad F. Baikpour M. Safari S. Saadat S.. (2016). Neural stem/progenitor cell transplantation for spinal cord injury treatment; a systematic review and meta-analysis. Neuroscience 322, 377–397. doi: 10.1016/j.neuroscience.2016.02.034 26917272

[ref41] Zhang H. A. Yuan C. X. Liu K. F. Yang Q. F. Zhao J. Li H.. (2022). Neural stem cell transplantation alleviates functional cognitive deficits in a mouse model of tauopathy. Neural Regen. Res. 17, 152–162. doi: 10.4103/1673-5374.314324, PMID: 34100451 PMC8451553

[ref42] Ziemba A. M. Gilbert R. J. (2017). Biomaterials for local, controlled drug delivery to the injured spinal cord. Front. Pharmacol. 8:245. doi: 10.3389/fphar.2017.00245, PMID: 28539887 PMC5423911

[ref43] Zipser C. M. Cragg J. J. Guest J. D. Fehlings M. G. Jutzeler C. R. Anderson A. J.. (2022). Cell-based and stem-cell-based treatments for spinal cord injury: evidence from clinical trials. Lancet Neurol. 21, 659–670. doi: 10.1016/S1474-4422(21)00464-6, PMID: 35569486

[ref44] Zyoud S. H. Smale S. Waring W. S. Sweileh W. M. Al-Jabi S. W. (2019). Global research trends in microbiome-gut-brain axis during 2009–2018: a bibliometric and visualized study. BMC Gastroenterol. 19:158. doi: 10.1186/s12876-019-1076-z, PMID: 31470803 PMC6716890

